# The investigation of serum phenylalanine levels based on infant feeding method: a cross-sectional study of children less than two years old with phenylketonuria (PKU)

**DOI:** 10.1186/s13006-024-00617-0

**Published:** 2024-02-13

**Authors:** Zaniar Mohammadzadeh, Loghman Sharifi, Asadolah Fatholahpour, Elham Bazshahi

**Affiliations:** 1 Department of Community Nutrition, Besat Hospital, Kurdistan University of Medical Sciences, Pasdaran St., Head On Hotel Shadi, Sanandaj, 66177-13446 Iran; 2 Non-Communicable Diseases Research Center, Alborz University of Medical Sciences, Karaj, Iran; 3 Department of Pediatrics, Faculty of Medicine, Kurdistan University of Medical Sciences, Sanandaj, Iran

**Keywords:** Phenylketonuria, Metabolic, Breastfeeding, Children

## Abstract

**Background:**

Clinical advice may suggest discontinuing breastfeeding after the diagnosis of phenylketonuria in infants as the only effective way to monitor the newborn's intake and accurate measurement of phenylalanine (Phe). This study aims to investigate the prevalence and duration of breastfeeding, as well as its effect on serum Phe levels in infants with phenylketonuria at Education and Therapy Medical Center, Be'sat Hospital, Iran.

**Methods:**

We conducted a cross-sectional study of 34 children under two years old diagnosed with phenylketonuria between September 2018 and December 2022. Infants were categorized as breastfed and non-breastfed (bottle-fed) based on their feeding method after diagnosis. Data on age at diagnosis, medical records, demographic information, and anthropometric indices were collected, and infants with incomplete data or mixed feeding (formula + breast milk) were excluded from the study.

**Results:**

Of 94 infants managed in our hospital, 34 had complete medical records. Among the all patients 13 (38%) continued to be breastfed combined with phenylalanine-free amino acid-based protein substitute, while 21 (62%) were did not receive breast milk. The mean duration of breastfeeding was 2.57 ± 0.59 (1–3) months. The mean age at diagnosis was 22.6 ± 18.4 days. Phenylalanine concentrations at diagnosis were mean 10, SD 5.44; range 4–24 mg/dL [0.22–1.33 μmol/L] in the breastfed group and mean 14.3, SD 10.2; range 5–37 mg/dL [0.27–2.05 μmol/L] in the non-breastfed group.Non-breastfed infants had lower serum Phe levels than breastfed infants: mean 3.76, SD 2.10; range 1–7 mg/dL [0.05–0.38 μmol/L] and mean 4.89, SD 3.68; range 2–19 mg/dL [0.11–1.05 μmol/L], respectively, although not statistically significant [(t (34) = 118.0, *P* = 0.51]. Also we found no significant associations in body measurements for weight, height, and head circumference at birth and final assessment.

**Conclusions:**

In conclusion, during treatment, there were no statistically significant associations between breastfeeding and serum Phe levels with growth in children with phenylketonuria.

## Background

Phenylketonuria (PKU) is an autosomal recessive inborn error of Inherited Metabolic Disorders (IMDs) that lead to enzymatic deficiencies within specific metabolic pathways caused by genetic mutations in the phenylalanine hydroxylase (PAH) gene encoding phenylalanine hydroxylase [1–3], which results in the inability to convert Phe to tyrosine, leading to increased phenylalanine concentrations in the blood and central nervous system [4]. Classic symptoms of untreated PKU include mental retardation, learning difficulties, spasticity, seizures, developmental delay, and congenital heart disease [5].

Comprehensive screening of newborns worldwide helps in the early identification and treatment of these metabolic disorders, subsequently reducing morbidity and mortality. A diagnostic method involving a heel prick test is conducted 24 h after birth to diagnose PKU [6]. Different methods for detecting PKU in dried blood spot sampling include fluorometric and colorimetric methods [7], enzymatic method [8], high-performance liquid chromatographic (HPLC) [9], and new techniques such as Tandem Mass Spectrometry [10]. Blood samples of all neonates in Iran are collected on days 3 to 5 after birth through a national program for screening and prevention of PKU established in 2007. Confirmation of PKU is done using by a colorimetric method, and if positive (phenylalanine levels of 4 mg/dL or higher [0.22 μmol/L]), infants are referred to be confirmed by the HPLC method [11]. Positive cases are referred to specialists for management and genetic counseling. Newborns with Phe levels equal to or greater than 4 mg/dL undergo regular follow-up, and if their Phe concentrations exceed 7 mg/dL (0.38 μmol/L), a restricted diet is initiated [12]. Since the inauguration of newborn screening in Iran, the incidence of PKU has been reported to be about one in 7000 live births, and this is noted to be more prevalent in some areas of the country [13]. Clinical sub-categories range from mild hyperphenylalaninemia (HPA) (Phe levels 120–360 μmol/ L) to the most common and severe form, classical PKU, defined as Phe > 1200 μmol/L [14]. Before newborn screening for PKU, Clinically, untreated disorder is characterized by irreversible intellectual disability, microcephaly, seizures, aberrant behavior, psychiatric symptoms, motor disturbances, and eczematous rash. Impairment of cerebral function had already occurred before newborn screening and treatment [15].

IMDs concerning lifelong management and treatment often focus on limiting substrate that cannot be metabolized from the diet and replacing other nutrients with supplements (medical foods) and drugs [16, 17]. Treatment of Infants diagnosed with PKU requires a unique low-phenylalanine formula, and it is recommended strict dietary therapy during their entire lifetime [18, 19]. However, in the management of infants with some IMDs, such as certain aminoacidopathies and urea cycle disorders, breastfeeding may be safely incorporated due to its lower protein and amino acid content than infant formula. Breastfeeding is included in management guidelines for some IMDs, including glutaric aciduria type I [20], maple syrup urine disease [21], phenylketonuria (PKU) [22, 23]; propionic acidemia [24], urea cycle disorders [25], and very long chain acyl-CoA dehydrogenase deficiency [26]. Since the Phe content in breast milk cannot be converted into tyrosine in the liver by the phenylalanine hydroxylase enzyme, unfortunately, exclusive feeding with breast milk in the first six months of life affects the cognitive-neural development of infants with PKU [27]. Severe cognitive impairments will be prevented with treatment [28]. In childhood, executive functions, such as working memory and reasoning/planning, attention, and processing speed, are mainly observed deficits [29]. Previously, the standard of care for patients with PKU was immediate cessation of breastfeeding to maintain adequate Phe levels with the combination of standard commercial infant formulas and amino acid-based protein substitutes without phenylalanine. In 1980, with the discovery of lower Phe levels in human breast milk compared to standard commercial infant formulas, breastfeeding began to replace the standard commercial formula in the protein-restricted diet of patients with PKU [30, 31]. Today, breastfeeding is encouraged in people with PKU [32, 33].

Previous studies reported that breastfed infants with PKU had no significant differences in weight gain, daily Phe intake, and mean serum Phe concentrations compared to bottle-fed infants with PKU [33–35]. On the other hand, a study by Banta-Wright et al. [32] showed that the mean serum Phe level in breastfed infants was lower than in bottle-fed infants with PKU.

In this study, we aimed to determine the prevalence and duration of breastfeeding, compare the effect of breastfeeding or non-breastfeeding on serum Phe levels, and anthropometric indices in infants with PKU. This is a subject for which limited results have been reported previously in the literature, and no study has been done to investigate this comparison in Iran.

## Methods

The Strengthening the Reporting of Observational Studies in Epidemiology (STROBE) guidelines were followed in conducting this study [36], and are available as a supplementary file. This cross-sectional study surveyed children below two years old with phenylketonuria in Iran. The Kurdistan University of Medical Sciences reviewed and approved all study procedures on January 4, 2023 (IR.MUK.REC.1401.306). All participants provided written informed consent.

Medical records were analyzed from September 2018 to December 2022 for patients with classic PKU (serum Phe level above 6 mg/dl at diagnosis) admitted to Education and Therapy Medical Center, Be'sat Hospital, located in the capital city of Kurdistan. Patients with incomplete records or insufficient data were excluded from the study.

### Study population

**Fig. 1:**
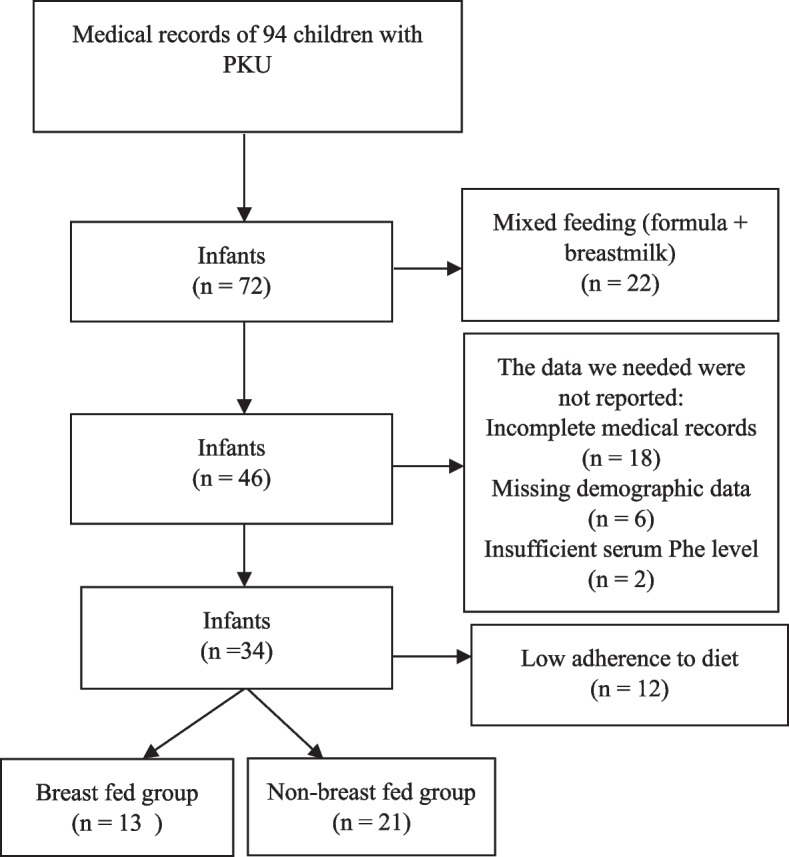
Flow chart illustrating the study selection process

The medical records of 94 children with PKU admitted to our center were analyzed retrospectively. The study included only patients under two years of age (from September 2018 to December 2022) who were examined at least once a month. Infants with incomplete medical records (age at diagnosis, anthropometric data, feeding information, and serum Phe level), missing demographic data (parental consanguinity, gross annual household income, maternal education level, place of residence), and low adherence to diet (patients who were not fed according to diet list and patients who were fed both breast milk, commercial formula, and phenylalanine-free amino acid based protein substitute) were excluded from the study (Fig. 1). Thirty-four of the subjects had complete medical records and were enrolled in the study.

### Setting

Infants were categorized as breastfed and non-breastfed (bottle-fed) according to the type of feeding after PKU diagnosis. Infants fed a combination of commercial formula and phenylalanine-free amino acid-based protein substitute after the diagnosis of PKU were included in the non-breastfed group. Infants who continued to be breastfed along with the phenylalanine-free amino acid-based protein substitute after the diagnosis of PKU were included in the breastfed group. After each breastfeeding, a phenylalanine-free amino acid-based protein substitute was given to the breastfed group. In the non-breastfed group, a combination of commercial formula and phenylalanine-free amino acid-based protein substitute was served at each feeding. All patients had their diet lists adjusted monthly and the volume of phenylalanine-free amino acid-based protein substitutes and commercial formulas were revised. Those whose consumptions were not monitored by their mothers (referred to as not adhering to the diet) were excluded. Also, infants who consumed mixed feedings (breast milk and formula) were not included in the study.

### Data collection

Demographic data according to the information provided by the family and medical records (age at the time of diagnosis, gender, parental consanguinity, gross annual household income, maternal education level, place of residence) and clinical and laboratory findings (based on physical examination, clinical and dietitian's records of infants who were assessed at least once a month; birth and final assessment of anthropometric indices, duration of breastfeeding, serum Phe level) of the patients were documented. Missing data on medical records or absence of serum Phe values recorded at least once a month were defined as insufficient medical records and were determined as exclusion criteria. The HPLC method was used to measure phenylalanine concentrations in blood samples. In the analysis of mean Phe levels, data were collected from the medical records of the newborn screening program and confirmatory diagnostic serum Phe levels, which were ≥ 6 mg/dl (≥ 0.33 μmol/L) for each patient. All data for the study was obtained by physicians and dietitians who worked at Be’sat Hospital.

### Data analysis

Statistical data analysis was performed using SPSS computer software version 15.0 (SPSS, Chicago, IL). To examine the normality of parameters, the Kolmogorov–Smirnov test was carried out. While categorical data were expressed as numbers and percentages (%), continuous data were expressed as mean ± standard deviation, median, full range, and 25^th^-75^th^ centiles. Categorical variables (gender, parental consanguinity, gross annual household income, place of residence, maternal education level, breastfeeding experience of the mother) were assessed using chi-square. Continuous variables (serum Phe levels, anthropometric indices) were evaluated using the Independent Samples t Test and Mann–Whitney U Test, respectively. A *p*-value < 0.05 was considered significant.

## Results

The study enrolled thirty-four infants diagnosed with PKU. Of these, 19 (56%) were female and 15 (44%) were male. The mean age at diagnosis was 22.5 ± 18.4 days (3–115). A comparison of breastfed and non-breastfed infants with PKU revealed no significant difference regarding gender and age at the time of diagnosis. There was a significant difference between the two groups regarding maternal education level t (3) = 34.4, *P* = 0.03 (Table 1).

**Table 1:** Demographic characteristics of infants with phenylketonuria

Variable		Breastfed group (*n* = 13)					Non- breastfed group (*n* = 21)							
	Ks test	Mean ± SD	P25	Median	P75	Range (minimum–maximum)	Mean ± SD	P25	Median	P75	Range (minimum–maximum)	DF	P^*^	test value
Diagnosis age (day)	0.001	23.8 ± 22.4	11.5	20.0	30.0	108 (7–115)	20.5 ± 9.7	14.5	20.0	30.0	37 (3–40)	34	0.97	135.5
Plasma Phe level at diagnosis (mg/dL)	0.004	10 ± 5.44	6.50	8.20	12.8	20 (4–24)	14.3 ± 10.2	7.00	11.2	17.2	32 (5–37)	34	0.18	99.0
Last Phe level (mg/dL)	0.001	4.89 ± 3.68	3.10	3.90	5.30	17 (2–19)	3.76 ± 2.10	2.80	3.80	4.55	6 (1–7)	34	0.51	118.0
Age at initiation of dietary therapy (d)	0.001	99.3 ± 147.76 (16–456)	20.0	30.0	124.0	440 (16–456)	65.5 ± 84.2 (10–366)	30.0	30.0	53.5	356 (10–366)	34	0.80	129.5
Age at the time of metabolic control (d)	0.001	131.31 ± 146.92 (46–486)	52.0	65.0	155.0	440 (46–486)	98.5 ± 83.9 (40–396)	60.0	65.0	96.0	356 (40–396)	34	0.83	130.5
Time from initiation of diet to metabolic control (d)	0.001	31.6 ± 3.12 (30–40)	30.0	30.0	33.0	10 (30–40)	31.6 ± 2.2 (30–35)	30.0	30.0	34.0	5 (30–35)	34	0.80	130.5
Gender (%)												1	0.60	0.273
Male		5 (38.5)					10 (47.6)							
Female		8 (61.5)					11 (52.4)							
Parental consanguinity, n (%)												1	0.85	0.035
Yes		6 (46.2)					9 (42.9)							
No		7 (53.8)					12 (57.1)							
Income (%)^**^												1	0.052	5.93
Low		3 (23.1)					12 (57.1)							
Moderate		6 (46.2)					8 (38.1)							
High		4 (30.8)					1 (4.8)							
Place of residence, n (%)												1	0.08	2.93
Urban		12 (92.3)					14 (66.7)							
Rural		1 (7.7)					7 (33.3)							
Maternal education level, n (%)												3	0.03	34.4
No education		2 (15.4)					5 (23.8)							
Primary school		1 (7.7)					6 (28.6)							
Diploma		7 (53.8)					8 (38.1)							
University		3 (23.1)					2 (9.5)							
Initial postpartum		10 (76.9)					21 (100)					3	< 0.001	34.0
< 1month		2 (15.3)					0							
> 1month		1 (7.8)					0							
Duration of Breastfeeding, n (%)												3	< 0.001	34.0
< 1 month		1 (7.6)					21 (100)							
6 month		3 (23)					0							
12 month		4 (30.7)					0							
24 month		5 (38.7)					0							

The *p*-value is considered significant at < 0.05

*KS* Kolmogorov–Smirnov, *SD* standard deviation, *DF* degrees of freedom, *p25* 25th percentile, *p75* 75th percentile. Data are presented as means and standard deviation, median (minimum–maximum), range, and 25th -75th centiles

^*^P values result from Mann–Whitney U test for continuous variables and χ2 test for categorical variables

^**^Income levels were classified based on the salary of the head of household in three categories (low: < 50,000,000 Rial, moderate: 50,000,000–1,50,000,000 Rial, and high: > 1,50,000,000 Rial)

Table 2 shows the anthropometric indices of patients for each group. No significant differences were seen in body measurements for weight, height, and head circumference at birth and final assessment. However, the average anthropometric indices of infants at birth and at the end of the evaluation in both groups were within the normal ranges of the Iranian standard growth centile charts [37] (Table 3). The weight, height and head circumference centiles of Iranian children were similar to UK and USA values [38].

**Table 2:** Anthropometric results for infants with phenylketonuria

Variable		Breastfed group (= 13)					Non- breastfed group (n = 21)							
	KS	Mean ± SD	P25	Median	P75	Range (minimum–maximum)	Mean ± SD	P25	Median	P75	Range (minimum–maximum)	DF	P^*^	test value
Age at the time of metabolic control (d)	0.001	131.31 ± 146.92 (46–486)	52.0	65.0	155.0	440 (46–486)	98.5 ± 83.9 (40–396)	60.0	65.0	96.0	356 (40–396)	34	0.83	130.5
Birth weight (kg)	0.200	3.14 ± 0.41	2.85	3.11	3.35	1 (3–4)	3.04 ± 0.48	2.65	3.20	3.50	2 (2–4)	32	0.53^*^	-0.633
last weight (kg)	0.157	10.5 ± 2.54	8.10	11.0	12.7	8 (6–14)	10.1 ± 3.09	7.90	10.8	13.5	10 (4–14)	32	0.72^*^	0.362
Birth height (cm)	0.199	48.8 ± 3.31	45.5	50.0	51.5	11 (43–54)	49.1 ± 3.41	47.0	49.0	50.0	16 (44–60)	32	0.77^*^	-0.28
Last height (cm)	0.041	75.8 ± 8.67	69.0	77.0	83.5	25 (61–86)	75.9 ± 8.93	68.5	80.0	82.5	32 (56–88)	34	0.97^**^	136.0
Birth head circumference (cm)	0.001	34.2 ± 3.00	32.0	33.0	36.0	11 (31–42)	33.9 ± 1.49	33.0	34.0	34.5	6 (31–37)	34	0.72^**^	125.0
Last head circumference (cm)	0.020	44.4 ± 2.10	43.5	45.0	47.0	8 (39–47)	44.5 ± 3.23	42.0	45.0	47.0	12 (37–49)	34	0.95^**^	124.5

The *p*-value is considered significant at < 0.05

*KS* Kolmogorov–Smirnov, *SD* standard deviation, *DF* degrees of freedom, *p25* 25th percentile, *p75* 75th percentile. Data are presented as means and standard deviation, median (minimum–maximum), range, and 25th -75th centiles

^*^P values result from Two Independent Sample T-tests

^**^P values result from Mann–Whitney U test

**Table 3:** References of weight, height and head circumference for birth in Iranian infants

Variable	Boys (*n* = 15)	Girls (*n* = 19)
	Mean ± SD	Mean ± SD
Birth weight (kg)	3.24 ± 0.42	3.12 ± 0.42
Birth height (cm)	50 ± 2.2	49.5 ± 1.2
Birth head circumference (cm)	35 ± 1.4	34.21 ± 2.3

Data are presented as means and standard deviation

### Prevalence and duration of breastfeeding

After the diagnosis of PKU, breastfeeding was continued in 13 (38%) infants combined with a phenylalanine-free amino acid-based protein substitute. In 21 (62%) infants, mothers stopped breastfeeding and continued to feed with the combination of commercial formula and phenylalanine-free amino acid-based protein substitute. The mean duration of breastfeeding was 14.3 ± 0.59 (range 1–24) months (Table 1). No other relationship was detected between other demographic characteristics (gross annual household income, place of residence, first breastfeeding experience of the mother) and the duration of breastfeeding.

### Analysis of serum Phe levels

Serum Phe concentrations of non-breastfed infants [mean 3.76, SD 2.10; range 1–7 mg/dL (0.05–0.38 μmol/L)] were lower than that of breastfed infants during the breastfeeding period [mean 4.89, SD 3.68; range 2–19 mg/dL (0.11–1.05 μmol/L)]. However, there was no significant difference in serum Phe levels between the two groups receiving breast milk and bottle t (34) = 118.0, *P* = 0.51.

## Discussion

In this study of thirty-four infants with PKU, we found no differences in phenylalanine concentrations between the breastfed and non-breastfed infants and no differences in anthropometric indices.

Several studies have discussed using human milk or breastfeeding in the dietary management of infants with PKU. Adequate growth and development of infants with PKU in these studies supports the efficacy of using human milk [39–41]. Breastfeeding also emphasizes the benefits of strengthening the emotional bond between mother and child and accepting the disease. Maintaining regular feeding with breast milk as a source of Phe in the treatment of PKU means that phenylketonuria infants can receive all the benefits of breast milk, even if they receive it as part of mixed breastfeeding [41].

Lower serum Phe levels in most breastfed infants compared to non-breastfed infants have been investigated previously, which shows the effect of breastfeeding on serum Phe levels in patients with PKU [34, 42, 43]. On the other hand, a recent study revealed similar serum Phe levels in breastfed and non-breastfed patients with PKU [32]. Although no significant relationship was found in our research, the level of Phe concentrations was lower in the formula-receiving group. Even though no significant difference was detected in comparing both groups, the bottle-fed group kept all Phe values within the reference range of the United States and European guidelines. The treatment target of blood Phe concentrations is between 120–360 μmol/L [1.38 to 4.14 mg/dL] for all patients aged 0–12 years [44, 45]. It is worth emphasizing that breastfeeding in our population is performed without precise daily measurements, while daily feeding with commercial formula is measured accurately. This difference in the amount consumed and the inaccuracy of measuring breast milk can affect the serum level of phenylalanine.

We consider that the breastfeeding duration of children with PKU over the 24 months of follow-up was 2.57 ± 0.59 months. In this regard, in a study by Rijn et al. [43], breastfeeding duration was 2.5 months. However, Demirkol et al. [35], McCabe et al. [41], and Motzfeldt et al. [31] found mean breastfeeding duration in phenylketonuric patients of 6.1, 8.9, and 7.0 months, respectively. Although the breastfeeding duration of observation was below that recommended by the World Health Organization, one should take into account the fact that, when traditional treatment is used, children are definitively weaned during the first month of life. Maintaining breast milk in these phenylketonuria infants' diets benefits them for a more extended period.

The time for blood Phe levels to reach normal levels from treatment is crucial. In this study, we observed that the results of the two groups were statistically similar. No differences were found between the two groups for birth weight, age at initiation of dietary therapy, or blood Phe level. This suggests that the breastfed and non-breast-fed groups were comparable at the beginning of treatment. The time to achieve metabolic control was similar, proving that the breastfeeding strategy did not adversely affect initial control.

To the best of our knowledge, this is the first study of Iranian infants with PKU conducted to compare the serum levels of Phe in two groups. Also, previous studies on anthropometric indicators only examined body weight while we assessed the height growth in our patients. Height growth is a better indicator to assess long-term nutritional status.

### Limitations

There are several limitations to the present study. Firstly, it is important to acknowledge the small sample size, which limits the ability to make an optimal comparison between the two groups. Of the 94 cases managed in our hospital, only 34 were analyzed. Additionally, only the most compliant families attended sufficiently regularly to be included. It is possible that monthly attendance signified compliance, which may have been reflected in adherence to diet and infant feeding recommendations. This self-selection of participants may have engendered both a selection and a collider bias [46]. It should also be noted that various parameters, such as infant medications, infections, treatment compliance, and socio-cultural conditions, which were not accounted for in our study, could have potentially influenced both growth and serum Phe levels. Finally, maternal factors such as maternal medicines, maternal age, employment, and smoking status, which are associated with breastfeeding, were not evaluated in our study. The small sample size precluded adjusted analyses. This is a single-site study, which limits generalization of findings.

## Conclusion

Our conclusion was that PHE levels did not differ between breast milk and non-breast milk groups. Using breast milk as a source of Phe in PKU treatment can be achieved. with proper control of blood Phe levels and infant growth within normal limits. Positive reinforcement of the emotional bond between mother and baby is an inherent behavioral benefit of breastfeeding. This positively impacts the acceptance of the condition and compliance with treatment.

## Data Availability

The corresponding author confirms that authors will make the data (in de-identified form, if human data) used in the manuscript, code book, and analytic code available to editors and readers upon request either before or after publication for checking.

## Abbreviations

HPLC: High-performance liquid chromatographic
IMDs: Inherited Metabolic Disorders
Phe: Phenylalanine
PKU: Phenylketonuria
